# A systematic exploration reveals the potential of spermidine for hypopigmentation treatment through the stabilization of melanogenesis-associated proteins

**DOI:** 10.1038/s41598-022-18629-3

**Published:** 2022-08-25

**Authors:** Sofia Brito, Hyojin Heo, Byungsun Cha, Su-Hyun Lee, Sehyun Chae, Mi-Gi Lee, Byeong-Mun Kwak, Bum-Ho Bin

**Affiliations:** 1 Department of Applied Biotechnology, Ajou University, Suwon, 16499 Republic of Korea; 2 Department of Biological Sciences, Ajou University, Suwon, 16499 Republic of Korea; 3 Biosolution Co, Seoul, Republic of Korea; 4 Neurovascular Unit Research Group, Korean Brain Research Institute, Daegu, 41062 Republic of Korea; 5 Bio-Center, Gyeonggido Business and Science Accelerator, Suwon, 16229 Republic of Korea; 6 School of Cosmetic Science and Beauty Biotechnology, Semyung University, Chungbuk, 27136 Republic of Korea

**Keywords:** Molecular biology, Protein transport, Computational biology and bioinformatics

## Abstract

Spermidine (SPD), a polyamine naturally present in living organisms, is known to prolong the lifespan of animals. In this study, the role of SPD in melanogenesis was investigated, showing potential as a pigmenting agent. SPD treatment increased melanin production in melanocytes in a dose dependent manner. Computational analysis with RNA-sequencing data revealed the alteration of protein degradation by SPD treatment without changes in the expressions of melanogenesis-related genes. Indeed, SPD treatment significantly increased the stabilities of tyrosinase-related protein (TRP)-1 and -2 while inhibiting ubiquitination, which was confirmed by treatment of proteasome inhibitor MG132. Inhibition of protein synthesis by cycloheximide (CHX) showed that SPD treatment increased the resistance of TRP-1 and TRP-2 to protein degradation. To identify the proteins involved in SPD transportation in melanocytes, the expression of several solute carrier (SLC) membrane transporters was assessed and, among 27 transporter genes, SLC3A2, SLC7A1, SLC18B1, and SLC22A18 were highly expressed, implying they are putative SPD transporters in melanocytes. Furthermore, SLC7A1 and SLC22A18 were downregulated by SPD treatment, indicating their active involvement in polyamine homeostasis. Finally, we applied SPD to a human skin equivalent and observed elevated melanin production. Our results identify SPD as a potential natural product to alleviate hypopigmentation.

## Introduction

Hypopigmentation disorders result in the appearance of white macules in the skin. These anomalies may occur due to a decrease in the number of melanocytes in the body, their inability to produce melanin, or abnormal transfer of mature melanosomes to neighboring keratinocytes^1^. Hypopigmentation may be diffused or localized, acquired or congenital, and is associated with a specific distribution pattern^2^. Acquired disorders include vitiligo, pityriasis alba, tinea versicolor and postinflamatory hypopigmentation, while congenital disorders include albinism, piebaldism, tuberous sclerosis and hypomelanosis of Ito. In addition, premature hair graying is a condition characterized by the early appearance of gray hair, generally before the age of 20 years in Caucasians and before 30 years in African Americans^3^. Though benign, these conditions can cause discomfort and affect the psychological state of the individual, and also increase UV sensitivity. In the cosmeceutical sector, melanogenesis modulators derived from natural sources are typically more appealing to customers. Several treatments for hypopigmentation include steroids, oral drugs, phototherapy and transplants, raising concerns for the safety and commodity of these methods^4^. For example, topical corticosteroids used to treat vitiligo can cause skin atrophy^5,6^. Selenium sulfide 2.5%, used to treat pityriasis versicolor can cause contact dermatitis^7,8^. Also, imidazoles and triazoles can commonly cause gastrointestinal disturbances, such as nausea and vomiting, when treated orally^9,10^. Therefore, it is important to develop new hypopigmentation treatments that are more natural and human friendly.

Polyamines are ubiquitous positively charged amines found in all living organisms^11^. These molecules are formed by the reaction of two or more amino groups, and can easily bind with various negatively charged macromolecules, including DNA, RNA, proteins, and acidic phospholipids^12,13^. As a result, they play essential roles in cell growth, proliferation, differentiation, gene regulation, immunity, as well as in the synthesis of proteins and nucleic acids. Spermidine (SPD) is one of the most prevalent natural mammalian polyamines. This molecule is synthesized from putrescine (PUT) or by oxidative degradation of spermine (SPM). In the polyamine pathway, the precursor ornithine initially undergoes decarboxylation by ornithine decarboxylase to generate the diamine PUT. Afterwards, the triamine SPD and the tetraamine SPM are formed by spermidine synthase and spermine synthase reactions, respectively. Moreover, intracellular SPD levels are dependent on polyamine uptake from the extracellular space, endogenous biosynthesis, catabolism, and excretion^14^. SPD plays important roles in cellular biochemical functions, including nucleic acid and protein synthesis, structure maintenance, and stability. It is estimated that approximately 13% of SPD is bound to DNA, and approximately 57% is bound to RNA to stimulate and improve protein synthesis^15^. Despite the capacity of an organism to produce SPD by de novo biosynthesis, external supplementation is essential to maintain a balanced polyamine pool^16^. Plant-derived foods, such as whole grains, vegetables, and legumes contain high levels of SPD, as do aged cheese and raw animal tissues^17–19^. Moreover, tissue SPD concentrations typically diminish with aging^20^. As a result, SPD supplementation exerts anti-aging effects and increases the overall lifespan in several animal models, including nematodes, flies, rodents, and humans^21^. More specifically, SPD can reduce cancer-associated mortality, enhance autophagy, ameliorate mitochondrial biogenesis, induce intestinal maturation, and improve function of the cardiovascular system, skeletal muscle, the immune system, liver, kidneys, and nervous system.

Melanogenesis is a complex process that regulates pigmentation. Melanin biosynthesis is tightly regulated by melanocyte-specific enzymes: TYR, tyrosinase-related protein 1 (TRP-1) and TRP-2^22,23^. TYR is a bifunctional enzyme that catalyzes the hydroxylation of l-tyrosine to l-3,4-dihydroxyphenylalanine (l-DOPA), followed by the oxidation of l-DOPA to l-dopaquinone. Moreover, TRP-2 functions as a dopachrome tautomerase that converts dopachrome to its carboxylate derivative, 5,6-dihydroxyindole-2-carboxylic acid (DHICA), whereas TRP-1 functions as a DHICA oxidase that converts DHICA to a carboxylated indole-quinone that forms eumelanin (a brown-black pigment)^24,25^. Following synthesis and subsequent processing in the endoplasmic reticulum and Golgi apparatus, melanogenesis proteins are trafficked to melanosomes for pigment formation^26^. Subsequently, tyrosinase degradation occurs spontaneously, which is dependent on the ubiquitin proteasome and on the endosomal/lysosomal systems^26,27^. While practically all proteins are affected by such mechanisms, diminished stability and function of tyrosinase has severe implications on pigmentation. Therefore, improving the stability of tyrosinase and its associated proteins constitutes a promising strategy to overcome pigmentation defects. Since SPD has suggested to interact with proteins and improve their stability^28–30^, we aimed to find whether this polyamine could be a potential natural compound to target hypopigmentation.

In this study, we analyzed the potential of SPD for hypopigmentation treatment through a systematic exploration approach which revealed changes in protein degradation. Subsequently, we analyzed the role of SPD in protein stability and, as polyamine transporter expression can vary according to cell type, we also aimed to identify the main SPD transporter present in melanocytes. Lastly, we investigated the potential of SPD application in vivo through a human skin equivalent model.

## Results

### SPD treatment increases melanin production

**Figure 1:**
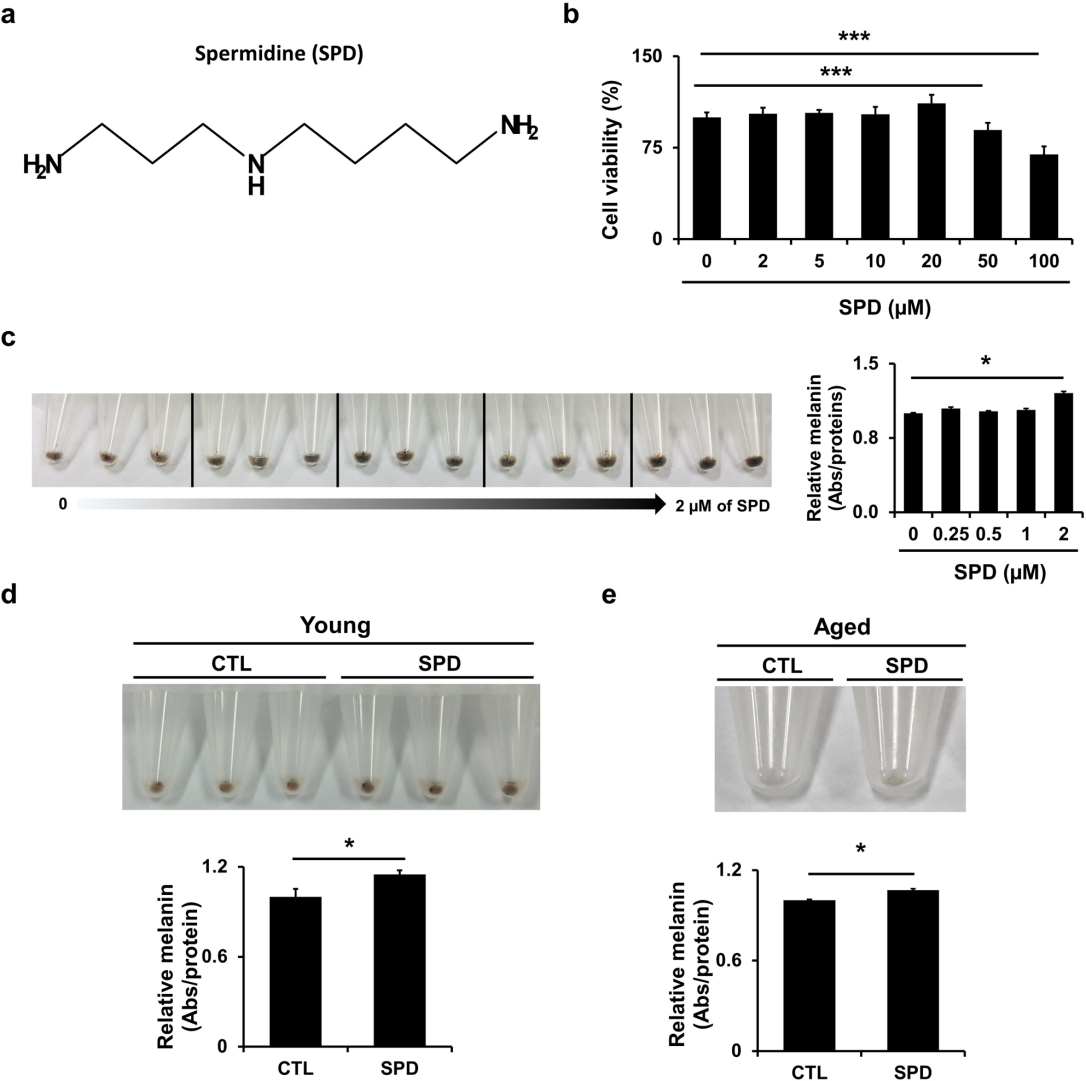
Spermidine (SPD) treatment increases melanin production. (**a**) Scheme of the chemical structure of SPD. (**b**) Cell viability of MNT-1 cells was evaluated using 3-(4,5-dimethylthiazol-2-yl)-2,5-diphenyltetrazolium bromide (MTT) assays after 24 h of SPD treatment. (**c**) Melanin levels in MNT-1 cells treated with 0, 0.25, 0.5, 1, and 2 μM of SPD for 10 days were measured at 450 nm. The data are representative of two independent experiments. (**d**,**e**) Melanin levels in young and aged primary human melanocyte cells treated with 1 μM of SPD for 10 days were measured at 450 nm. The data are representative of three independent experiments. CTL: control, *P < 0.05, **P < 0.01*,* ***P < 0.005.

SPD is a polyamine compound (C_7_H_19_N_3_) that exerts various metabolic functions in living organisms (Fig. 1a). To investigate the effect of SPD on melanogenesis, normal human primary melanocytes and a human melanoma MNT-1 cell line, with the capacity to produce all stages of melanosomes, were obtained. The cytotoxic effects of SPD were evaluated by treating MNT-1 cells with various concentrations of SPD (Fig. 1b). It was confirmed that cell viability was affected only at concentrations of 50 and 100 μM. Therefore, in this study, SPD was administered in concentrations below these values. Quantification of melanin content in MNT-1 cells treated with increasing concentrations of SPD revealed increased melanin production in these cells (Fig. 1c). Moreover, based on a previous report indicating that SPD exerts distinct effects in young vs. aged tissues^31^, young and aged primary human melanocytes were treated with SPD. SPD increased melanin production in both cell types (increments of 15 ± 5% and 12 ± 2% in young and aged cells, respectively) (Fig. 1d,e).

### SPD treatment modulates genes involved protein degradation

**Figure 2:**
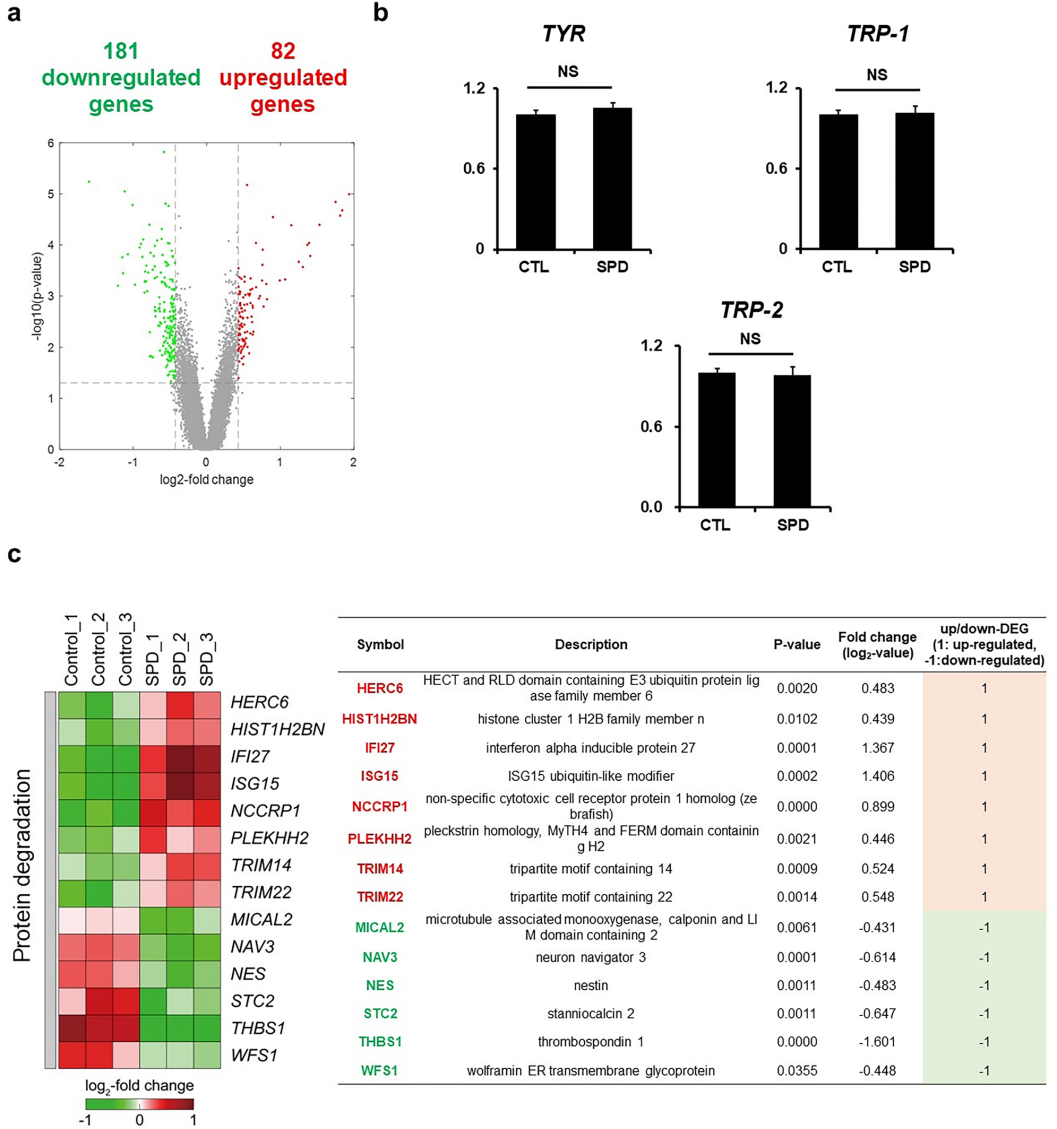
A systematic exploration reveals that SPD treatment modulates genes involved protein degradation. (**a**) RNA-sequencing analysis of 1 µg of total RNA of primary melanocytes after treatment with 1 μM of SPD for 7 days revealing the upregulation (red) and downregulation (green) in gene expression levels. (**b**) mRNA expression levels of melanogenesis-related genes of primary melanocytes after treatment with 1 μM of SPD for 10 days. The data represent the results of three independent experiments. CTL: control, NS: non-significant. (**c**) RNA-sequencing analysis of 1 µg of total RNA of primary melanocytes after treatment with 1 μM of SPD for 7 days revealing the upregulation (red) and downregulation (green) in expression levels of genes involved in protein degradation.

To further understand the molecular implications of SPD treatment, the effects of this polyamine were analyzed through a systematic approach. Firstly, RNA sequencing performed in SPD-treated cells revealed the modulation of only 181 downregulated and 82 upregulated genes (Fig. 2a), of which melanogenesis-related genes were not included. To further confirm this, we next analyzed the effects of SPD on the expression levels of tyrosinase family genes *TYR*, *TRP-1* and *TRP-2*, which tightly regulate melanogenesis (Fig. 2b). mRNA expression levels confirmed that this polyamine did not alter melanogenesis-associated gene expression. However, we found that several of the genes whose activity was altered by SPD were related to protein degradation (Fig. 2c). Several of the altered genes are involved in ubiquitination, a system for degradation of proteins that is also associated with the degradation of melanogenesis proteins. Altogether, these data infer that SPD does not increase melanogenesis by upregulating its associated genes' expression levels, but suggests it might influence melanogenesis by modulating protein stability.

### SPD treatment improves the stabilities of TRP-1 and TRP-2

**Figure 3:**
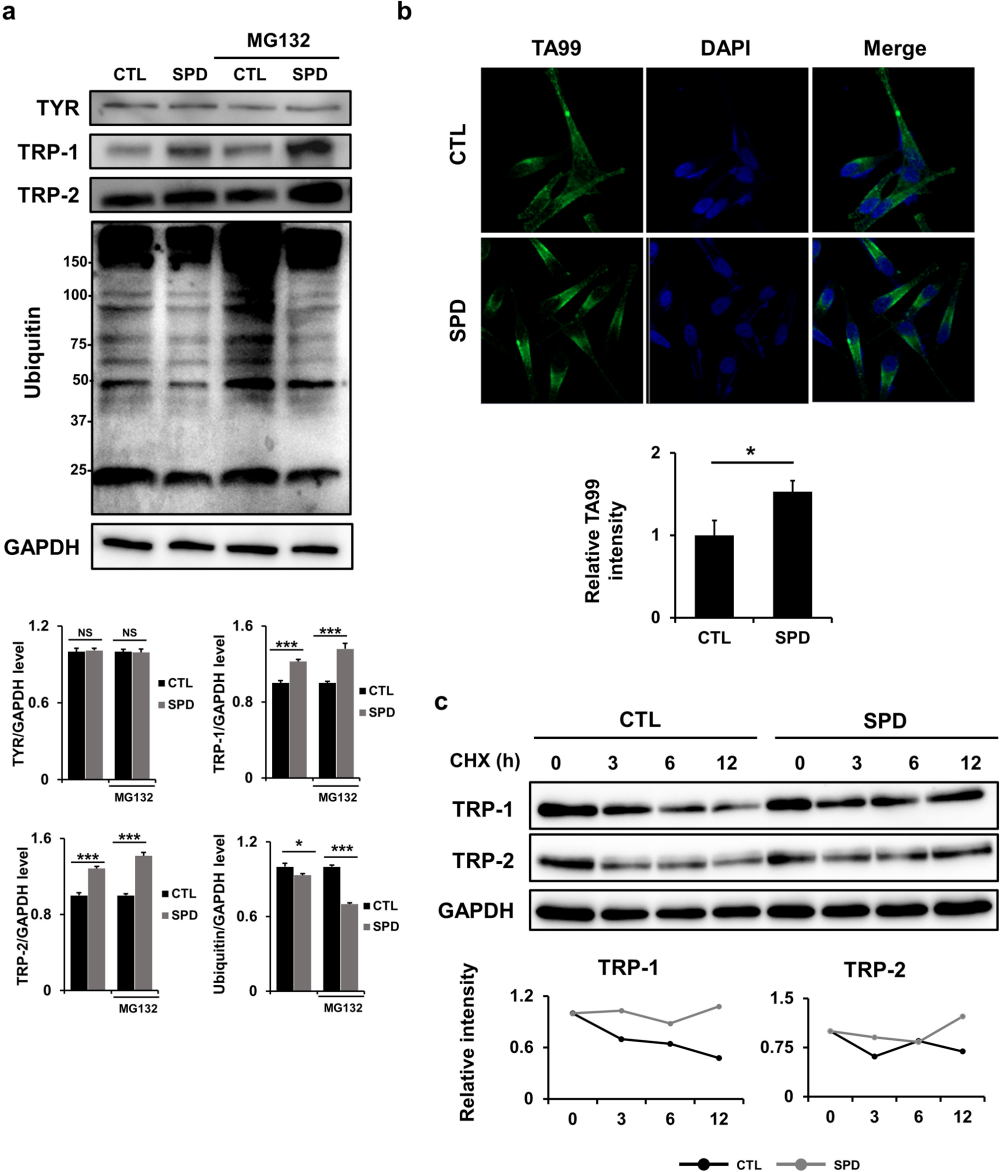
SPD treatment improves the stabilities of tyrosinase-related protein (TRP)-1 and TRP-2. (**a**) Western blots of B16F10 cells treated with 5 μM of SPD for 2 days and subsequent treatment with 10 μM of MG132 for 2 h. Samples were derived from the same experiment and blots were processed in parallel. Original blots are presented in Fig. S1. The data represent the results of three independent experiments. (**b**) TA99 protein levels examined by confocal microscopy at a magnification of 400× in MNT-1 cells treated with 2 μM of SPD for 10 days. Cell nuclei (blue) were stained with 4′,6-diamidino-2-phenylindole dihydrochloride (DAPI). (**c**) Western blotting of MNT-1 cells with treated with 10 μM of CHX for the indicated time periods and subsequent treatment with 2 μM of SPD. Samples were derived from the same experiment and blots were processed in parallel. Original blots are presented in Fig. S2. The data represent the results of three independent experiments. CTL: control, NS; non-significant, *P < 0.05, **P < 0.01*,* ***P < 0.005.

Since melanin production is regulated by a balance of synthesis and degradation of melanogenesis-associated proteins, we next aimed to better understand the effects of SPD at the protein level. SPD treatment resulted in a significant increase in TRP-1 and TRP-2 protein levels but not TYR (Fig. 3a). Moreover, since the proteasome system is involved in the degradation of melanogenesis-related proteins^32^, the expression of ubiquitin under SPD treatment was analyzed and revealed an altered ubiquitin expression pattern. This result, which is consistent with the RNA-seq data (Fig. 2c), may indicate that SPD increases melanogenesis by decreasing protein degradation via ubiquitination. Moreover, after treatment with SPD and subsequent application of the proteasome inhibitor MG132, expression levels of TRP-1 and TRP-2 were also significantly increased, unlike TYR, and ubiquitin expression decreased. These data further demonstrate that SPD modulates ubiquitination in addition to confirming that it has no impact on TYR expression. In addition, confocal microscopy revealed accumulation of the late-stage melanosome marker TA99 in cells treated with SPD (Fig. 3b), indicating the accumulation of highly pigmented melanosomes in these cells. Finally, to block the transcription of melanogenesis-related genes, cycloheximide (CHX) was applied (Fig. 3c). Under these conditions, SPD treatment improved the stabilities of TRP-1 and TRP-2, further demonstrating that SPD increases melanogenesis by improving protein stability which, in turn, increases TRP-1 and TRP-2 expression levels.

### Identification of putative SPD transporters in primary human melanocytes

**Figure 4:**
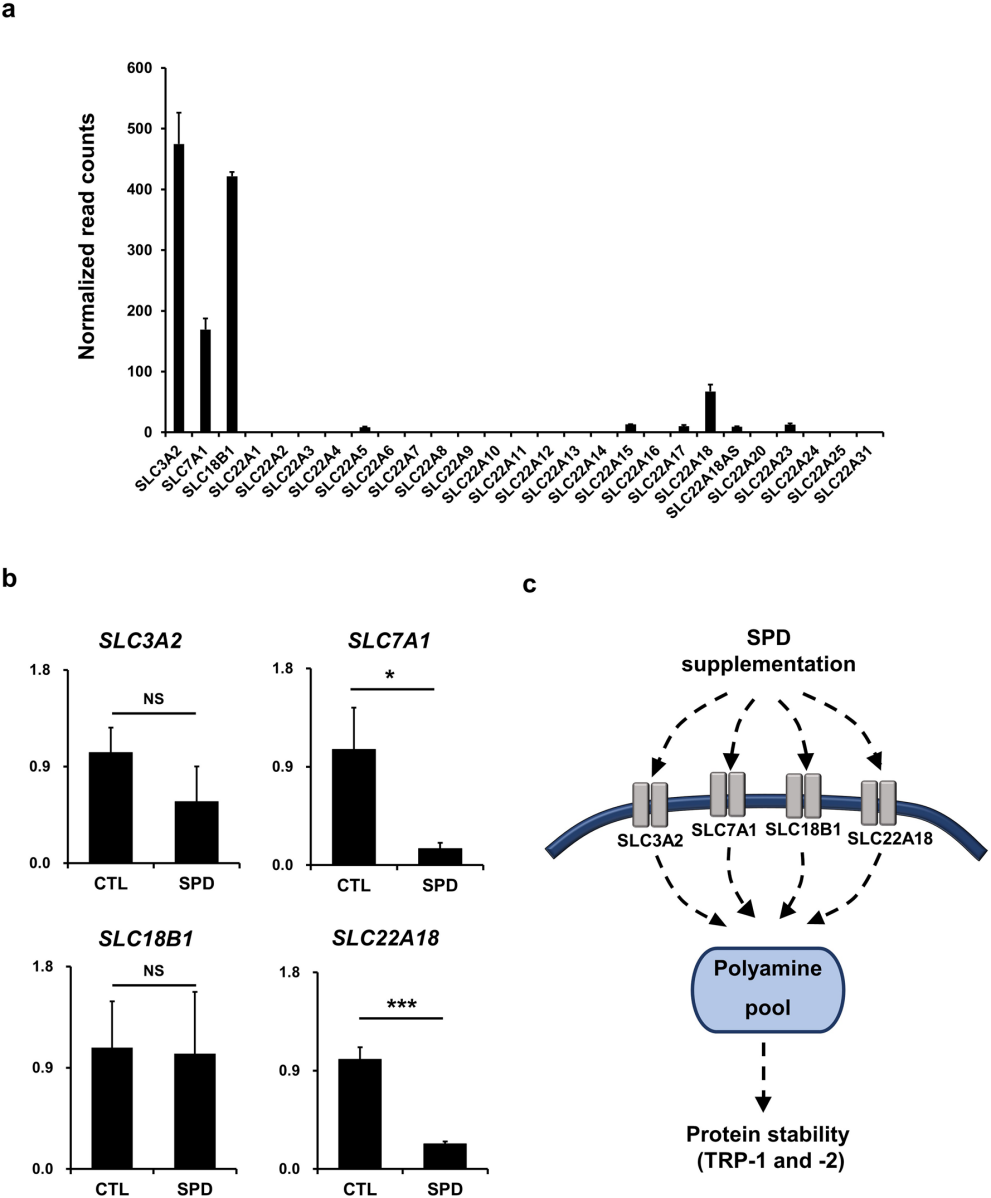
Identification of putative SPD transporters in primary human melanocytes. (**a**) RNA-sequencing analysis of 1 µg of total RNA of primary melanocytes representing the expression levels of solute carrier (SLC) family transporters. (**b**) mRNA expression levels of primary melanocytes after treatment with 1 μM of SPD for 10 days. The data represent the results of three independent experiments. CTL: control, NS: non-significant, *P < 0.05, **P < 0.01*,* ***P < 0.005. (**c**) Schematic representation of the effects of SPD treatment in the improvement of the stabilities of TRP-1 and TRP-2 through the increase of the cellular polyamine pool.

Intracellular polyamine levels are regulated by a combination of their synthesis, catabolism, and transport^33^. In order to identify the proteins involved in SPD transportation in melanocytes, the expression of several solute carrier (SLC) membrane transporters was assessed since they are reportedly involved in polyamine transportation^12,34–38^. Firstly, transcriptome data (Fig. 4a) revealed that, among the 27 transporter genes, *SLC3A2*, *SLC7A1, SLC18B1*, and *SLC22A18* were highly expressed in primary human melanocytes. Since the balance of a substrate is maintained due to the selective action of transporters, and transporter protein expression levels decrease under high substrate concentration as a strategy to maintain ideal cytosolic levels and avoid cytotoxic effects^39^, we next aimed to observe the mRNA expression levels of these three genes under SPD treatment (Fig. 4b). Interestingly, *SLC7A1* and *SLC22A18* were the only genes responsive to the treatment, indicating their active involvement in polyamine homeostasis. Hence, SPD supplementation leads to an increase of the cellular polyamine pool, mediated by the actions of *SLC3A2*, *SLC7A1, SLC18B1*, and *SLC22A18,* thus increasing the stability of TRP-1 and -2 (Fig. 4c). These data demonstrate the potential role these transporters play in polyamine homeostasis to support protein stability for melanogenesis.

### SPD increases melanogenesis in vivo

**Figure 5:**
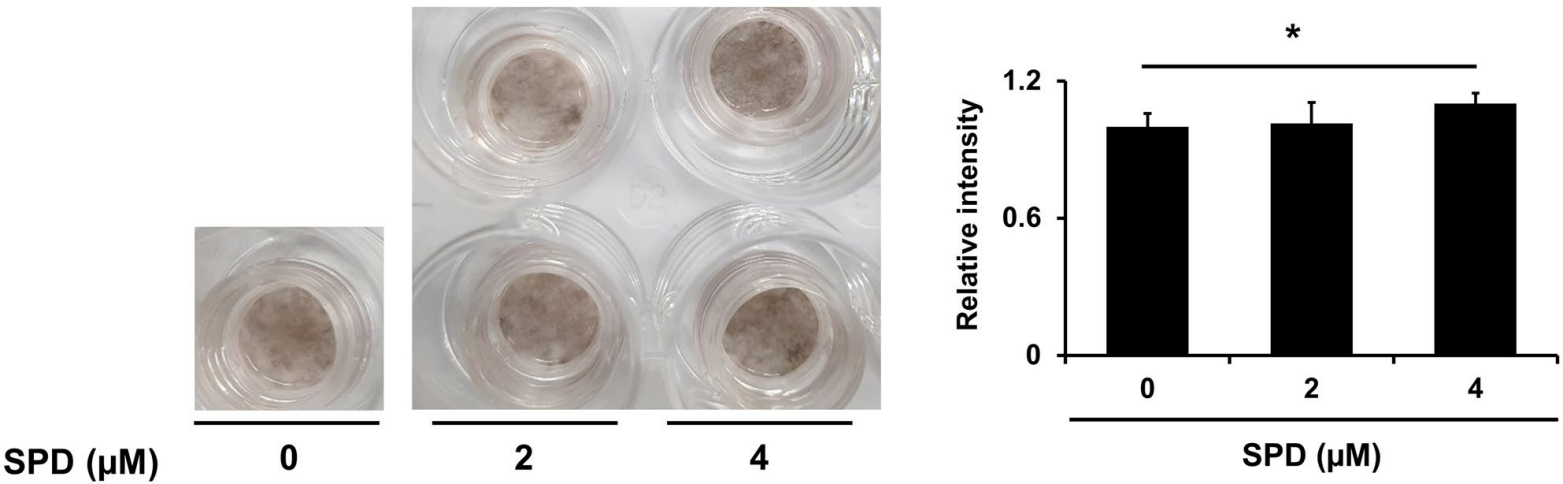
SPD increases melanin production in a human skin equivalent model. Treatment of a human skin equivalent model with 2 and 4 μM of SPD for 2 weeks. The data represent the results of three independent experiments. *P < 0.05, **P < 0.01*,* ***P < 0.005.

Finally, we aimed to analyze the potential of applying SPD as a treatment for hypopigmentation. We confirmed the effects of SPD in vivo by applying this polyamine to a human skin equivalent containing melanocytes. SPD supplementation resulted in a significant increase in melanin levels (increment of 10 ± 5%), confirming its potential application for treatment of hypopigmentation disorders (Fig. 5).

## Discussion

In the present study, a systematic exploration approach revealed that SPD treatment modulates genes involved in protein degradation. Furthermore, protein analysis demonstrated that the melanogenesis-related proteins TRP-1 and TRP-2 were stabilized following SPD treatment. Finally, a human skin equivalent model revealed that SPD increased melanin production in vivo. Therefore, our findings demonstrate the potential of SPD for ameliorating hypopigmentation.

The polyamine pool gradually declines with age^33^. A previous study analyzed polyamine levels in 14 different tissues of an aging mouse model and revealed that SPD levels dropped in 11 of the 14 tissues^40^. SPM decreased only in the skin, heart, and muscles, while PUT levels were remarkably low in all tissues and at all ages. Indeed, SPD plays important roles in many tissues, and SPD supplementation leads to the relief of several age-related conditions. Moreover, hypopigmented macules, such as stellate pseudoscars and idiopathic guttate hypomelanosis, are frequently observed in photodamaged skin of aged individuals, and hair graying is a common feature of aging^41^. With this in consideration, since aged cells have a deficient polyamine pool, it seems reasonable that SPD treatment can restore it to more optimal levels thus increasing melanogenesis. However, our results also demonstrate that SPD treatment led to melanogenesis improvement in young cells. The beneficial effects of SPD supplementation in young mouse models have also been previously reported^42^. Thus, this infers that the benefits of SPD treatment are not limited to aged individuals, despite the polyamine pool knowingly decreasing during aging, and may also target non-age-related hypopigmentation, or premature hair graying. Hence, we state that SPD is a promising treatment to alleviate hypopigmentation, in which therapy doses should be customized to each patient for maximum efficacy.

Melanogenesis in human skin is tightly regulated by the expression of melanin-associated genes and the subsequent enzymatic reactions caused by these genes. Our results revealed that SPD treatment improved melanogenesis without altering the expressions of melanogenesis genes. Instead, SPD altered the expression of genes related with protein degradation, including proteasome-associated genes. Simultaneously, we observed an improvement of the stability of the melanogenesis proteins TRP-1 and -2 and decreased ubiquitination after SPD application. Indeed, melanogenesis proteins are digested by the proteasome system^27,43,44^. Ubiquitination is a system that targets eukaryotic proteins for breakdown and is dependent on a cascade of E1 ubiquitin-activating enzymes, E2 ubiquitin-conjugating enzymes and E3 ubiquitin-protein ligases that subsequently bind the target protein for degradation in the proteasome^15^. SPD has been implied in the impairment of ubiquitination by inhibition of ubiquitin ligases^45^, thus this is a plausible explanation for why SPD stabilizes TRP-1 and TRP-2. Interestingly, SPD did not significantly affect TYR expression. A previous study that observed the degradation rates of TYR, TRP-1, and TRP-2 revealed that TYR has a greater half-life than TRP-1 and TRP-2^46^. Therefore, TYR may have a more rigid protein structure that confers it better stability. Another factor to take into consideration is that protein stability is also influenced by differences in the degradation machinery. Humans are estimated to possess 500–1000 E3 ubiquitin ligases, which are thought to play a key role in protein identification^47^. In this regard, it is also possible that TYR might be tagged by different ubiquitin ligases from those binding TRP-1 and TRP-2. Further research about the distinct structures of these proteins and their ubiquitin binding sites is necessary to answer these questions. Furthermore, fatty acids have been shown to regulate melanogenesis^48^. Similar to our findings, palmitic acid treatment of melanocytic cells led to decrease in ubiquitination, which increased the stability of melanogenesis proteins without alterations of their mRNA levels. However, in contrast with our work, palmitic acid treatment increased TYR expression, but not TRP-1 and -2. It is interesting to note that one of the molecular targets of SPD is lipid metabolism, which is regulated through autophagy^49^. Indeed, activation of autophagy has been linked with induction of melanogenesis and regulation of melanosome biogenesis in melanocytes^50^. Therefore, we cannot rule out the possibility that SPD might also alter the stability of melanogenesis proteins through modulation of autophagy. However, since our bioinformatics data did not identify changes in autophagy after SPD supplementation, we did not focus on that mechanism in this investigation. This may be due, in part, to the fact that we used a lower dose of SPD than other researches, which was based on the results of the cell viability assay. The modification of lipid metabolism following SPD treatment needs to be further investigated in order to fully comprehend these events. Nonetheless, our results infer that targeting protein stability could constitute a novel therapeutic strategy to overcome hypopigmentation.

Since polyamines are positively charged at physiological pH, they require a transport system to take up exogenous polyamines and/or eliminate excess polyamines from the cell^51,52^. Moreover, while the mechanisms involved in polyamine biosynthesis and catabolism have been thoroughly studied, the mammalian polyamine transport system is still poorly known. The SLC superfamily, which has 384 members, is the second-largest group of membrane transporter proteins in the human genome^53^. SLC transporters are primarily involved in the transportation of small molecules into cells by moving substrates via ion or electrochemical gradients like sodium or proton gradients^54^. Several SLC transporters have been implied in polyamine transportation^36^. Despite that, the SPD transporters in melanocytes had not been defined yet. We observed that *SLC3A2*, *SLC7A1*, *SLC18B1*, and *SLC22A18* transporters genes were highly expressed in melanocytes. Moreover, since substrate balance is maintained by the selective action of transporters, we hypothesized that SPD supplementation would downregulate their expression levels to maintain cytosolic levels. However, only *SLC7A1* and *SLC22A18* were responsive to SPD treatment. As a result, we can imply that these two proteins may be actively involved in SPD-dependent polyamine homeostasis in melanocytes. In future works, a more thorough analysis will be required to better define the SPD transporters in melanocytes. Cell-based transport assays are limited since various endogenous transporters can disturb the transportation of a target molecule. Instead, proteoliposome-based transport assay performed with a purified pure transporter offers an effective model system for investigating the transportation of a target molecule^55^. Prior to reconstructing a proteoliposome, expression and purification investigations should be addressed. These methodologies present the potential to complement our investigation and precisely identify the SPD transporters in melanocytes.

In conclusion, this study indicates that SPD is a promising compound for the treatment of hypopigmentation disorders through improved protein stability, thus demonstrating great economical value given the current market demand for natural and human-friendly cosmetic products.

## Materials and methods

### Cell culture and materials

Moderately pigmented normal human primary melanocytes (Cascade Biologics, Portland, OR, USA) were cultured in M-254 medium (Cascade Biologics) containing human melanocyte growth supplement (HMGS; Cascade Biologics) and antibiotics (100 μg/mL penicillin, 100 μg/mL streptomycin; Thermo Fisher Inc., Waltham, MA, USA). MNT-1, a malignant melanoma cell line, was kindly provided by Dr. Ai-Young Lee at Dongguk University, who originally received it as a gift from Dr. Vincent J. Hearing at the National Institutes of Health (Bethesda, Maryland, USA). B16F10 cells were obtained from the Korean Cell Line Bank (Seoul, Republic of Korea). MNT-1 and B16F10 cells were cultured in minimum essential medium (Thermo Fisher Inc.) containing 20% fetal bovine serum (Gibco, Carlsbad, CA, USA), antibiotics (100 μg/mL penicillin, 100 μg/mL streptomycin), 20 mM 4-(2-hydroxyethyl)-1-piperazineëthanesulfonic acid, and 10% Dulbecco's modified Eagle's medium/high glucose (Lonza, Basel, Switzerland). All cells were maintained at 37 °C and 5% CO_2_. SPD (Sigma-Aldrich, St. Louis, MO, USA) was dissolved in distilled water, and new stocks were prepared monthly. CHX and MG132 were purchased in Sigma-Aldrich and kept at − 30 °C until use.

### Cell viability assay

Cells were seeded at a density of 1 × 10^4^ cells/well in a 96-well plate (Corning, New York, USA). After overnight incubation, cells were treated with 0, 2, 5, 10, 20, 50, and 100 μM of SPD for 24 h. Subsequently, 100 μL of 3-(4,5-dimethylthiazol-2-yl)-2,5-diphenyltetrazolium bromide reagent was added to the medium in each well and incubated for 3 h at 37 °C. The reduced formazan crystals were solubilized in 50 μL of dimethyl sulfoxide (Sigma-Aldrich) and absorbances were measured at 570 nm in a microplate reader (SpectraMax 190 Absorbance Plate Reader; Molecular Devices, Sunnyvale, CA, USA).

### Melanin assay

Melanocytes were seeded at a density of 1 × 10^5^ cells/dish. After overnight incubation, cells were treated with SPD for 7 days. Cells were lysed in 0.5 M Tris–HCl (pH 7.5) containing 1% NP-40, 0.15 M NaCl, and 5 mM MgCl_2_, as previously described^56^. Cell lysates were centrifuged at 15,000 rpm and 4 °C for 20 min. The protein content of each supernatant was measured using a bicinchoninic acid (BCA) assay, and 100 μL of NaOH solution was added to dissolve each pellet and heated at 80 °C for 1 h. Melanin levels in the cells were determined by measuring absorbance at 450 nm. Absorbances were divided by the amount of protein used to determine each total melanin content.

### Western blotting analysis

Cells were lysed in a solution of 0.05 M Tris–HCl, pH 7.5, 0.15 M NaCl, and 0.01 M MgCl_2_. Cell lysates were centrifuged at 15,000 rpm and 4 °C for 20 min, and protein contents were quantified using a BCA assay. Aliquots of 20 μg of protein per well were separated in a sodium dodecyl sulfate–polyacrylamide gel, and electro-transferred from the gel to a polyvinylidene fluoride membrane (Sigma-Aldrich). After blocking with 2.5% bovine serum albumin for 1 h, membranes were incubated with primary antibodies targeting TYR (1:300; Thermo Fisher Inc.), ubiquitin (1:500; Santa Cruz Biotechnology, Dallas, TX, USA), TRP-1 (1:4000), TRP-2 (1:4000), and GAPDH (1:4000) (Abcam, Cambridge, UK).

### Immunofluorescence

Cells were cultured on Lab-Tek chamber slides (Nunc, NY, USA), as described previously^56^. Cells were stained with TA99 (Thermo Fisher Inc.) and 4′,6-diamidino-2-phenylindole (DAPI; Sigma-Aldrich). Secondary antibody staining was performed using Alexa Fluor 488–conjugated F(ab′)2 fragment of goat anti-mouse IgG antibody (Invitrogen, Waltham, Massachusetts, USA). Images were obtained using an LSM 800 confocal microscope (Carl Zeiss, Jena, Germany).

### RNA-sequencing analysis

Total RNA was isolated from saline- and SPD-treated melanocytes using RNeasy Mini Kit columns (Qiagen, Hilden, Germany), according to the manufacturer’s instructions. RNA quality was assessed using an Agilent 2100 bioanalyzer with an RNA 6000 Nano Chip (Agilent Technologies, Amstelveen, Netherlands), and RNA quantity was determined using an ND-1000 Spectrophotometer (NanoDrop Technologies, Inc., DE, USA). The RNA integrity number (RIN) values for all of the samples were larger than 9. Poly(A) mRNA isolation from the total RNA and subsequent fragmentation were performed the TruSeq Stranded mRNA LT Sample Prep Kit (Illumina, San Diego, CA, USA), according to the manufacturer’s instructions. The adaptor ligated libraries were sequenced using an Illumina NovaSeq 6000 (Macrogen, Inc., Seoul, South Korea). mRNA sequencing was performed for three biological replicates of each condition. From the resulting read sequences for each sample, adapter sequences (TruSeq universal and indexed adapters) were removed using the cutadapt software (version 2.7)^57^. The remaining reads were then aligned to the *Homo sapiens* reference genome (GRCh38) using TopHat2 software (version 2.1.1) with default parameters^58^. After the alignment, we counted the numbers of reads mapped to the gene features (GTF file of GRCh38.89) using HTSeq^59^. Read counts for the samples in each condition were then normalized using TMM (trimmed mean of M-values) normalization of the edgeR package^60^.

### Identification of differentially expressed genes (DEGs)

To identify DEGs, a statistical hypothesis test was performed^57^. For each gene, a T-statistic value was calculated using the Student’s t-test for comparison of saline- and SPD-treated melanocytes. An empirical distribution of the T-statistic value for null hypothesis was estimated by performing all possible combinations of random permutations of the samples. Adjusted *P*-values were obtained for each gene using a two-tailed Student’s t-test with an empirical null distribution. The DEGs were identified as genes with adjusted *P*-values ≤ 0.05, and absolute log_2_-fold changes > 0.428 (1.35-fold; the mean of the 0.5th and 99.5th percentiles of the null distribution of log2-fold changes).

### qPCR

Melanocytes treated with 1 μM SPD for 7 days were subjected to RNA extraction using a RNeasy Plus Mini Kit (Qiagen, Hilden, Germany). RNA concentrations were measured using a Nano-400A Micro Spectrophotometer (Allsheng, Zheijang, China). cDNA synthesis was performed using the AccuPower RT PreMix (Bioneer, Daejeon, Korea). cDNA, primer (F/R), and CYBR Q GreenBlue qPCR Master Mix (Cellsafe, Yongin, Republic of Korea) were dispensed into MicroAmp Fast Reaction Tubes (Applied Biosystems, Überlingen, Germany), and mRNA expression levels were determined using an AriaMx real-time PCR system (Agilent Technologies, Palo Alto, CA, USA).

### Human skin equivalent

A human skin equivalent model composed of primary human keratinocytes and melanocytes (KeraSkin™, Biosolution, Korea) was used. The basal layer of this human skin equivalent model is composed of dendritic cells that produce melanin granules. These cells produced pigmentation and became fully differentiated when cultured in an air–liquid interface culture for 2 weeks. Cells were then treated with 2 and 4 µM SPD for 2 weeks.

### Statistical analysis

Two-tailed Student’s t-test was used to analyze the differences between two groups.

## Supplementary Information

Supplementary Figure S1.

Supplementary Figure S2.

## Data Availability

The raw and processed RNA-seq data were deposited into the Gene Expression Omnibus (GEO) database with accession ID GSE209538.
