# Supplementary figures and images for: A systematic exploration reveals the potential of spermidine for hypopigmentation treatment through the stabilization of melanogenesis-associated proteins
Source: Sci Rep. 2022 Aug 25;12:14478. doi: 10.1038/s41598-022-18629-3 (PMC9411574; doi:10.1038/s41598-022-18629-3)

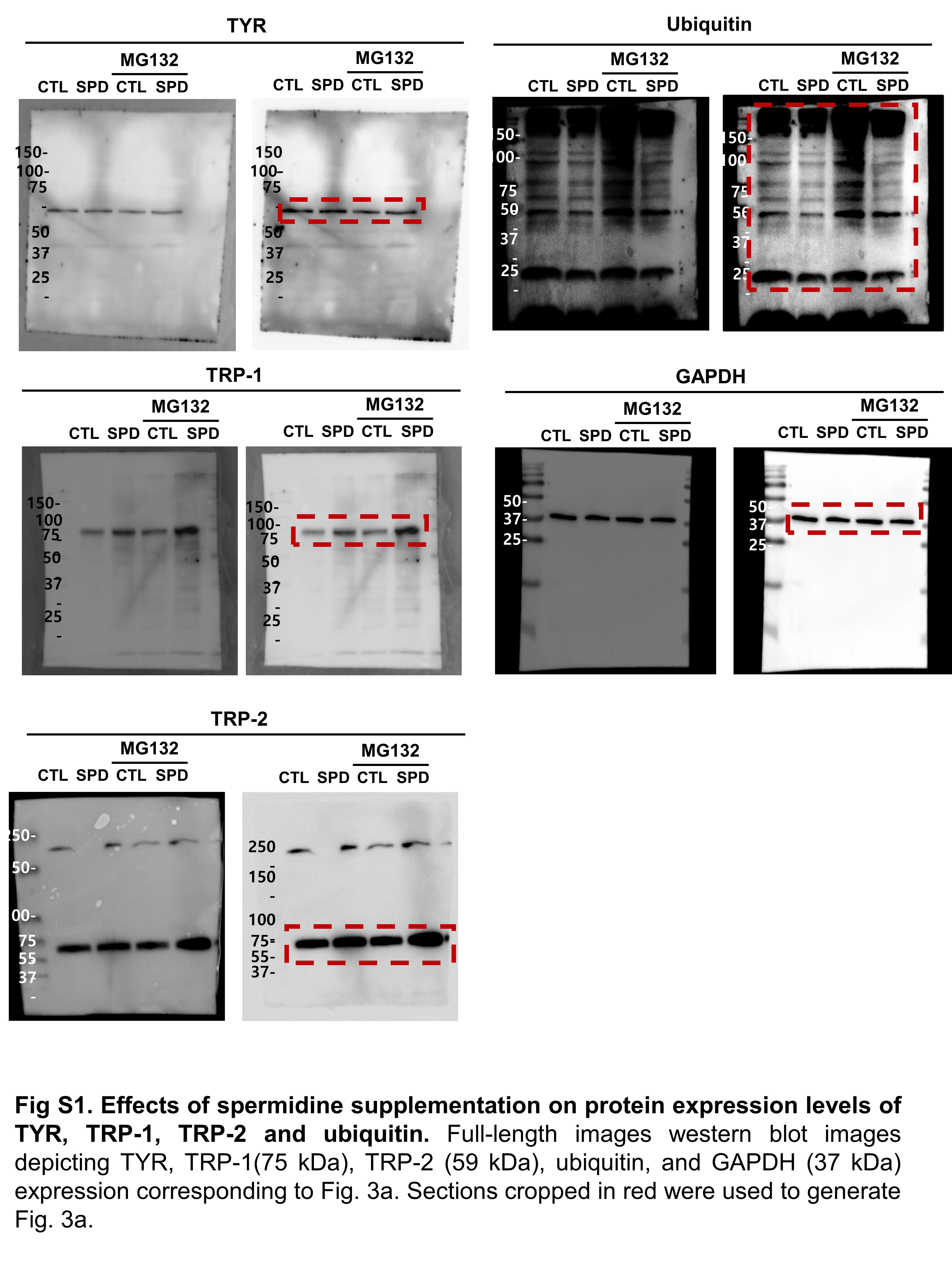

Supplement: Supplementary file 1 — Supplementary Figure S1. [file 41598_2022_18629_MOESM1_ESM.jpg]

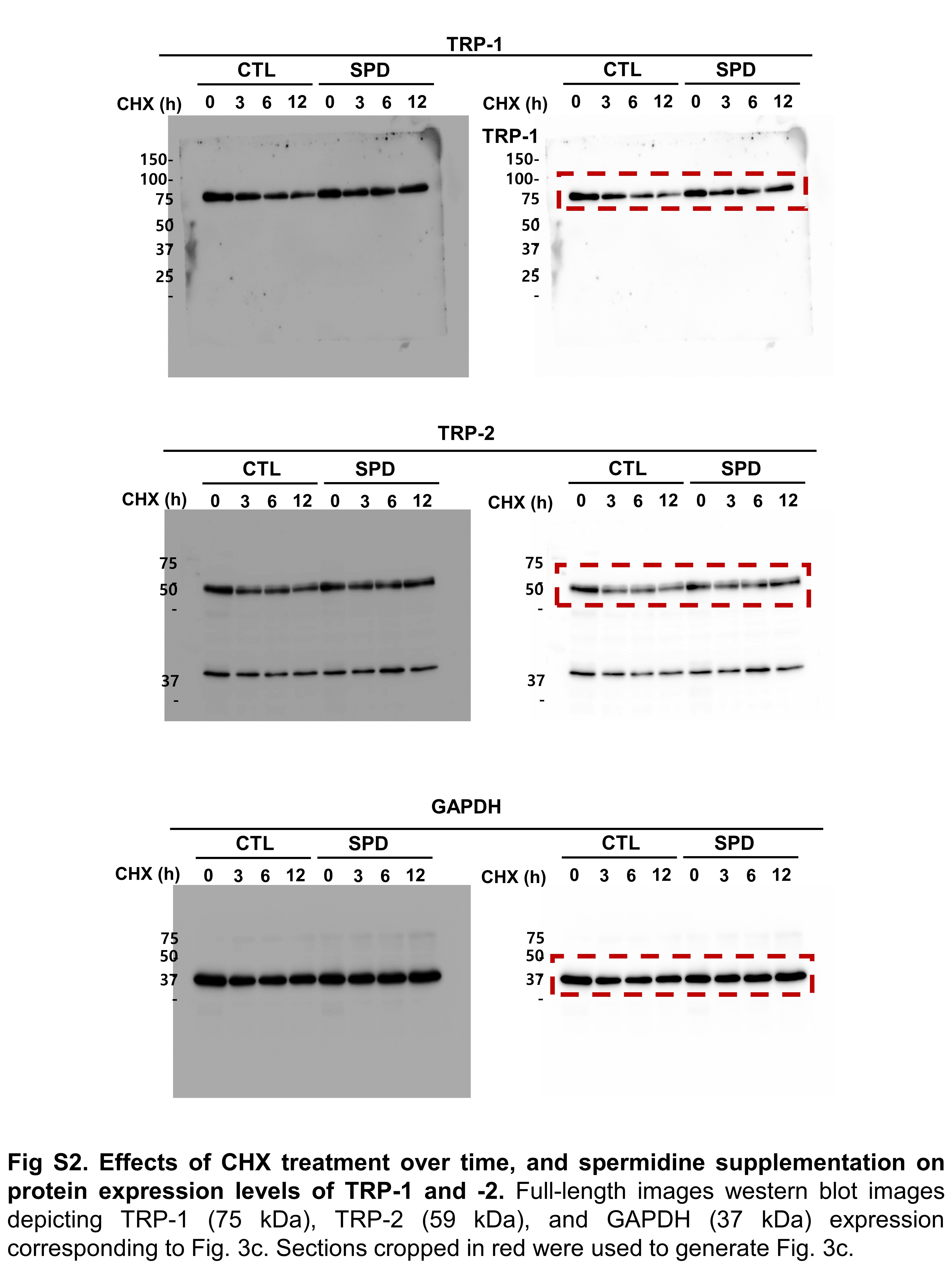

Supplement: Supplementary file 2 — Supplementary Figure S2. [file 41598_2022_18629_MOESM2_ESM.jpg]
